# Health risks and contamination degrees associated with heavy metals in three coastal fish from the Red Sea

**DOI:** 10.1038/s41598-025-15942-5

**Published:** 2025-09-01

**Authors:** Mahmoud Mahrous M. Abbas, Yasmine Mohamed EKraim, Abdullah S. Alnasser, Mohamed H. Ghanem

**Affiliations:** 1 Zoology and Entomology Department, Faculty of Science, Al-Azhar University, Cairo, 11884 Egypt; 2 Faculty of Natural Resources and Environmental Sciences, Department of Marine Resources, Omar El-Mokhtar University, Al Bayda, Libya; 3 Department of Civil Engineering, College of Engineering, Qassim University, 51452 Buraydah, Saudi Arabia

**Keywords:** Environmental assessment, Soaking treatments, Heavy metals, Carcinogenic and non-carcinogenic risks, Pollution mitigation, Ichthyology, Environmental impact

## Abstract

This study focuses on the levels of heavy metals (HMCs) in the commonly consumed marine fish from the Aqaba Gulf, Red Sea, Egypt. It evaluates the effectiveness of soaking treatments as a novel approach to reducing HMCs. The order of metals varied among species, with sigan and bongos fish following Mn < Ni < Cu < B < Fe < Zn, while mallas fish followed Mn < Ni < Cu < Zn < Fe < B. The highest level in untreated samples was observed in sigan for Zn (67.60 ± 2.34 µg/g ww-b), while the lowest was recorded in bongos for Mn (0.96 ± 0.07 µg/g ww-b). Soaking treatments significantly reduced HMCs in all species, as confirmed by environmental risk indices. Risk assessments revealed that the hazard index (HI-HMCs) values for children often exceeded the acceptable threshold of HI-HMCs ≤ 1, suggesting potential health risks despite reductions achieved through soaking. Among the soaking methods, the mixed soaked treatment, which combined salt and apple vinegar, showed the most pronounced reduction in metal levels, demonstrating a synergistic effect. In conclusion, while soaking treatments effectively mitigate HMCs contamination, further efforts are required to develop safer fish processing methods, particularly to safeguard vulnerable groups like children.

## Introduction

The Red Sea is renowned for its rich biodiversity, including a wide variety of commercially and ecologically significant fish species^1^. However, these valuable marine resources are increasingly threatened by the accumulation of heavy metals (HMCs), a critical environmental concern^2^. HMCs pollution in the Red Sea arises from multiple anthropogenic sources, including urban runoff, industrial effluents, emissions from desalination plants, maritime transport activities, and waste from fertilizer and clinker production facilities^3,4^.

Heavy metals are persistent environmental pollutants that tend to bioaccumulate, posing significant ecological and health risks due to their toxicity^5^. Based on their function in biological systems, HMCs can be divided into two categories. Low levels of essential HMCs, including copper (Cu), iron (Fe), nickel (Ni), zinc (Zn), and manganese (Mn), are biologically important in living systems. Non-essential HMCs, including lead (Pb), arsenic (As), mercury (Hg), cadmium (Cd), aluminium (Al), silver (Ag), and chromium (Cr) since they are hazardous even at low levels and have no recognised biological function in organisms^6–10^. Coastal and marine environments are particularly vulnerable to HMCs pollution, driven by human activities such as industrial discharge and urbanization^11^. Among the most concerning effects of this pollution is the bioaccumulation of toxic HMCs within marine ecosystems, which significantly endangers the health of Red Sea fish populations. This contamination not only threatens the sustainability of aquatic ecosystems but also jeopardizes the availability of essential resources, posing risks to both marine biodiversity and human consumers who rely on these species as a primary food source^5^.

Fish, as a primary protein source, hold significant nutritional and economic value^12^. However, they also serve as bioindicators of HMCs contamination due to their high trophic level and their tendency to accumulate pollutants in their tissues^13,14^. Elevated HMCs in fish can pose serious health risks to humans through dietary exposure, including cardiovascular, neurological, and reproductive disorders^15^. The persistence and bioaccumulation potential of HMCs, combined with their ability to disrupt enzymatic activities and induce carcinogenic or teratogenic effects, further emphasize the need to address this environmental challenge^16^. Recent studies have highlighted the evaluating the health and environmental risks posed by HMCs, particularly their impact on fish consumers and the health of humans^10,17–21^.

Despite global awareness, effective mitigation of HMCs in seafood remains a challenge, necessitating innovative approaches to minimize contamination and protect public health^21,22^. Among the various strategies, the use of natural and food-grade solutions such as saline and acidic treatments (e.g., vinegar) has gained attention for their potential to reduce HMCs residues in fish^23^. Organic acids like vinegar can form complexes with metals, thereby reducing their bioavailability and toxicity. Previous studies demonstrated that soaking seafood in acidic or saline solutions can significantly lower HMCs, providing a practical and accessible method for enhancing food safety^16,23,24^. Therefore, this study aimed to (1) Evaluate the levels of HMCs (B, Fe, Ni, Zn, Cu, and Mn) in three commercially significant fish muscles, *sigan,* bongos, and mallas fish from the Aqaba Gulf, Egypt. (2) Assess the efficacy of various treatments, including saline, acidic, and combined solutions, in reducing HMCs contamination. (3) Calculate the pollution and metal pollution indices (PI-HMCs and MPI-HMCs) to assess environmental contamination. (4) Use the health risk indices, including EDI-HMCs, HQ-HMCs, HI-HMCs, and CR-HMCs, to evaluate potential human health impacts.

## Materials and methods

### Sample collection and preparations

**Fig. 1:**
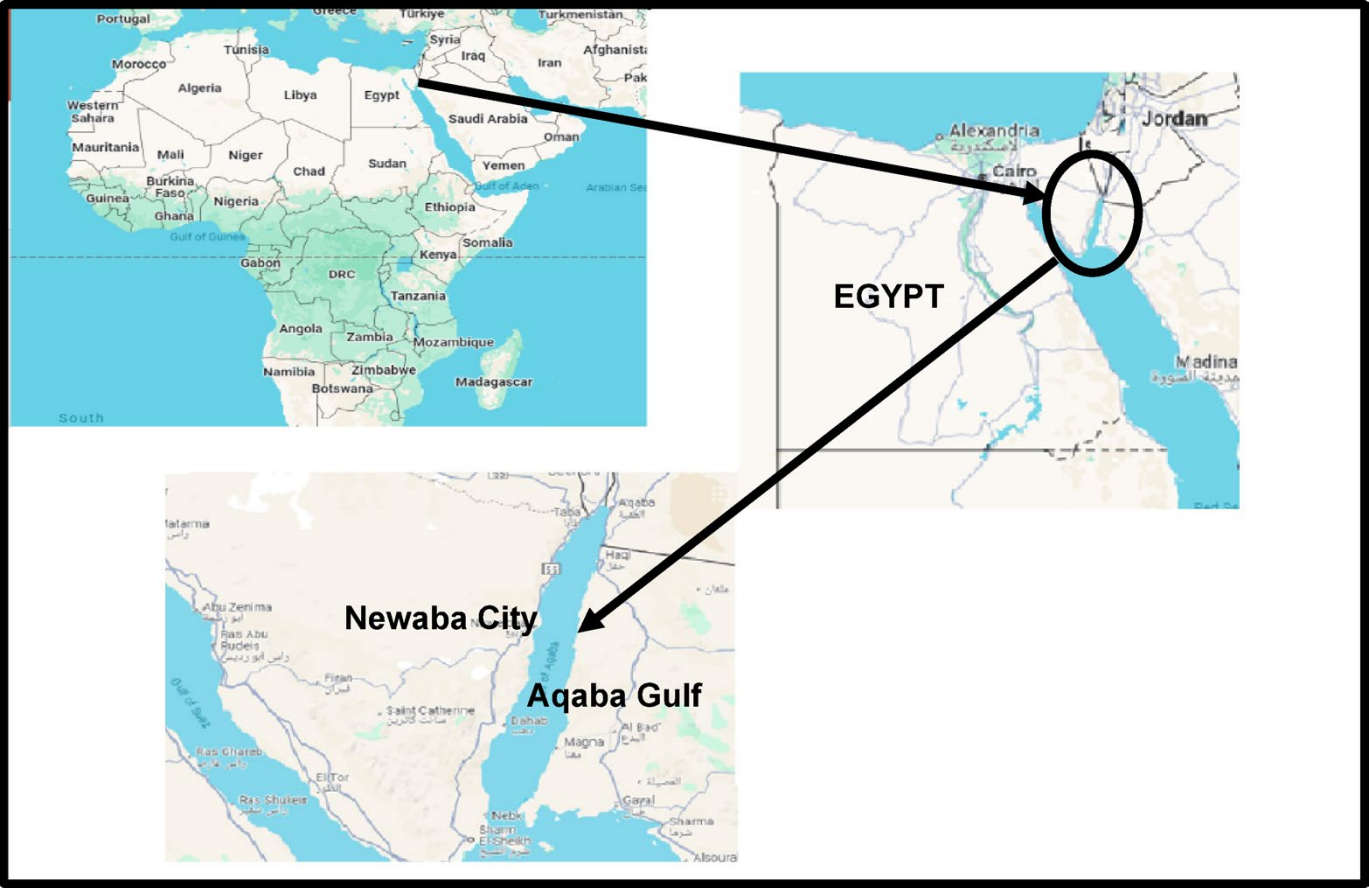
Map showing study area of Newaba City, Aqaba Gulf, Red Sea, Egypt.

Three species (120 fishes); the first is *Siganus luridus*, sigan fish in Arabic, which belongs to the Siganidae family and is primarily herbivorous, feeding on algae and seagrass (with an average weight of 166.14 ± 8.32 g and total length of 19.62 ± 1.18 cm); the second is *Lethrinus borbonicus*, bongos fish in Arabic, a member of the Lethrinidae family, which is a carnivorous species that feeds on crustaceans, molluscs, and small fish, playing an essential role as a predator in the ecosystem (with an average weight of 232.14 ± 12.32 g, and length of 21.63 ± 1.15 cm). Meanwhile, *Cheilinus lunulatus*, the third species, mallas fish in Arabic, belongs to the Labridae family and has an omnivorous diet, consuming invertebrates, small fish, and algae (with an average weight of 189.19 ± 13.81 g and total length of 20.63 ± 1.45 cm). Fish samples were collected from local fishermen from the Aqaba Gulf, Red Sea, Egypt, throughout September-December 2024. A sampling map was generated using QGIS version 3.22 (https://qgis.org) and the sampling area was marked using Microsoft PowerPoint and Paint (Fig. 1). A sterile plastic bag was used to individually pack each fish sample. To go to the Marine Biology Lab, Zoology Department, Faculty of Science, Al-Azhar University, Cairo, Egypt, the samples were labelled and shipped in an icebox at 4 °C. They were then kept frozen (− 18 °C) prior to preparation.

In lab, fish samples were washed, filleted, and to evaluate the effect of different solutions on the HMCs in the fillets of three fish species, the experiment was designed to include four primary samples (10 fish for each treatment). The first samples (untreated) involved soaking the samples in deionized water. The second samples (salt-soaked) used a salt solution with a level of 5% NaCl. The third samples (vinegar soaked) involved an apple vinegar solution with a level of 5%. In the fourth samples (mixed soaked), the samples were soaked in a mixture of the salt and vinegar-soaked solution in equal proportions. In each sample, the fillet was soaked at a 1:1 ratio (W/V) at room temperature for 30 min.

### HMCs measurements

According to AOAC^25^ with some modifications, about 0.5 g untreated, salt, vinegar, and mixed sample was placed in digestion tubes (50 mL) containing 5 mL of ultrapure HNO_3_ (65%) followed by 1 mL of H_2_O_2_ (30%), then stand overnight. After that, the tubes heated on a hot plate until complete digestion was achieved. Subsequently, the digested samples were cooled before being moved to a volumetric vessel (25 mL) and diluted using deionized water.

The iron, boron, copper, manganese, nickel, and zinc in diluted samples (untreated, salt, vinegar, and mixed soaked, n = 5) of the studied fish were measured using ICP-OES methods (Inductively coupled plasma optical emission spectrometry). However, the HMCs levels were calculated using the following Eq. (1):

1 $${\text{Metal}}\;{\text{concentration}}\;\left( {{\text{mg}}/{\text{kg}}} \right) = {\text{C}} \times {\text{V}} \div {\text{W}}$$

where C = concentration of the element in the digested solution (mg/L); V = final volume of the digested solution (L); W = weight of the tissue sample (kg, wet weight).

To confirm the precision and accuracy of the HMCs measurements, the National Institute of Standards and Technology (NIST) supplied a quality control sample, an external reference, and standard reference materials. The recovery rates for standard reference metals ranged between 90 and 110%. The internal reference was incorporated into each sample and standard solution. A series of diluted multi-meal standards were prepared, and the levels of HMCs in studied samples were ascertained using a five-point calibration curve. A calibration curve exhibiting an R^2^ value > 0.97 was utilized for level estimations. Additional quality assurance protocols encompassed reagent blanks and duplicate analyses. The limits of detection (LOD) values of HMCs were established at 0.0066 mg/kg for Ni, 0.0029 mg/kg for Zn, 0.025 mg/kg for Cu, 0.01 mg/kg for B, 0.0029 mg/kg for Mn, 0.0029 mg/kg for Fe, while the limits of quantification (LOQ) values were at 0.025 mg/kg for Ni, 0.0096 mg/kg for Zn, 0.084 mg/kg for Cu, 0.037 mg/kg for B, 0.0095 mg/kg for Mn, 0.0095 mg/kg for Fe. Sample levels were represented on a wet weight basis (ww basis) and quantified in mg/Kg.

### Environmental risk assessment

To determine the contamination degree of HMCs in aquatic organisms, different indices were employed^26^. The pollution index (PI-HMCs) and metal pollution index (MPI-HMCs) were used in this investigation to determine the degree of metal pollution.

#### PI-HMCs measurement

The PI-HMCs values were determined using the metal level of in fish muscles. It is found using this formula (2)^13^:

2 $${\text{PI-HMCs}} = {\text{L}}_{{\text{m}}} /{\text{L}}_{{{\text{bl}}}}$$

where L_m_ represents the metal level in muscles (mg/kg) and L_bl_ denotes the background levels of Zn, cadmium, Ni, iron, Cu, and lead^27^. The degree of contamination is clearly indicated by the PI-HMCs results. A PI-HMCs value of 1 or lower denotes very little pollution and minimum contamination. Low contamination is indicated by values between 1 and 2, which imply a slight presence of pollutants. A moderate level of contamination, denoted by a PI-HMCs range of between 2 and 3, indicates a discernible environmental impact. Significant environmental pollution is indicated by PI-HMCs values more than 3, which are classified as high contamination.

#### MPI-HMCs measurement

To compare the total level of HMCs between the different studied samples, the metal pollution index (MPI-HMCs) was calculated using Eq. (3)^28^:

3 $${\text{MPI-HMCs}} = \left( {{\text{L}}_{{1}} \times {\text{L}}_{{2}} \times {\text{L}}_{{3}} \times \cdots \times {\text{L}}_{{{\text{mx}}}} } \right)^{{{1}/{\text{n}}}}$$

where n is the HMCs number studied and L_1_, L_2_, L_3_, …, L_x_ are the metal levels in the studied samples (mg/kg). Higher MPI-HMCs values reflect greater toxicity of HMCs in that tissue. MPI-HMCs values between 10 and 20, indicate medium toxicity; between 5 and 10, low toxicity; between 2 and 5, very low toxicity; while below 2, no toxicity^29^.

### Health risk assessments

A methodology described by the USEPA^30^ was applied to determine the possible health risks linked to eating fish muscle tissue that includes metals. Based on the metal levels in the studied muscle, the EDI-HMCs and non-cancer and cancer risk indices were computed for this evaluation.

#### EDI-HMCs measurement

To evaluate exposure levels, the EDI-HMCs values, which is the average daily intake of a specific HMCs throughout a lifetime as a result of consuming fish muscle, was calculated. The EDI-HMCs is computed using the formula (Eq. 4) according to Mwakalapa^31^:

4 $${\text{EDI-HMCs}}\;{\text{values}} = \left( {\left( {{\text{EP}} \times {\text{IR}} \times {\text{FM}} \times {\text{ER}}} \right) \div \left( {{\text{BW}} \times {\text{AT}}} \right)} \right) \times {1}0^{{ - {3}}}$$

where the EP represents the duration of the exposure period (70 years), and the IR represents the fish consumption rate 62.25 g/day^32,33^; The metal level in fish muscle in μg/g ww-b is denoted by FM, 365 days per year is the exposure rate by ER, the body weights of consumers an adult weighs 70 kg, a young person 40, and a child weighs 15 kg), by BW, and the mean lifespan (365 days × 70 years = 25,550) by AT^30^.

#### THQ-HMCs measurement

To identify non-cancer health concerns related to consuming metal pollutants present in fish muscle tissue, the Target Hazard Quotient (THQ-HMCs) is employed. This measure evaluates the potential adverse health effects by comparing the EDI-HMCs of a HMCs to its RfD (oral reference dose). The Eq. (5) is used to determine the THQ-HMCs^34^:

5 $${\text{THQ-HMCs}}\;{\text{values}} = {\text{EDI-HMCs}}/{\text{RfD}}$$

where EDI-HMCs represents the metal’s estimated daily intake and RfD represents the oral reference dosage. The following are the RfD (mg/kg/day) values for the metals being studied: Lead 0.00357, Iron 0.7, Arsenic 0.003, Ni 0.02, Mn 0.14, Cu 0.04, Zn 0.3, and Barium 0.2^30^.

#### HI-HMCs measurement

The hazard index (HI) value, according to Cui^35^, is an additional computational calculation (Eq. 6) that adds up the THQ-HMCs values for the metals under study in order to show the effect of non-cancer risk.

6 $${\text{HI-HMCs}}\;{\text{values}} = \sum {\text{THQ-HMCs}}\;{\text{values}}$$

### Statistical examination

The statistical assessment was carried out using SPSS (Version 22). Levene’s test was employed to verify the homogeneity of variance. The Shapiro–Wilk test was employed to evaluate the data distribution’s normality. One-way ANOVA was applied to report statistically significant differences (*p* < 0.05) between the metals under study^36^. Additionally, Pearson’s correlation coefficient was performed to evaluate the relationships between the metal levels in the investigated fish. The statistical tables display the results as means ± standard deviation.

## Results and discussion

### Analysis of HMCs in fish and their soaked samples

**Fig. 2:**
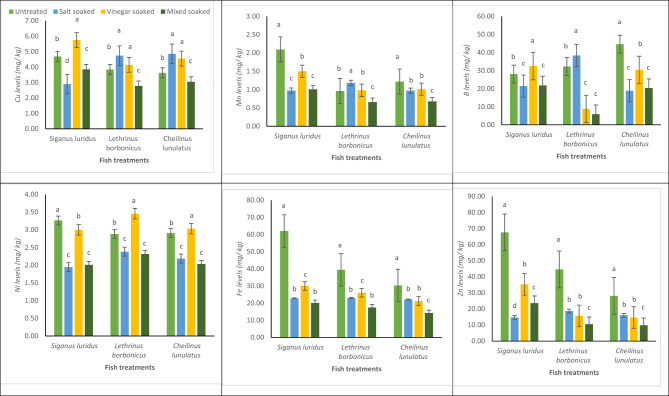
Impact of soaking treatments on HMCs (mg/kg ww basis, means ± SD, n = 5) in three marine fish fillets from Newaba City, Aqaba Gulf, Red Sea. *The differences in letters (*p* < 0.5, one-way ANOVA) show significant alterations in treatments for each species.

The iron, boron, copper, manganese, nickel, and zinc in untreated, salt, vinegar, and mixed soaked samples of the studied fish were represented in Fig. 2. However, the comparison of the levels of the studied metals with the previous studies was reported in Table 1.

**Table 1:** HMCs (mg/kg) in the studied species compared to different investigations.

	Fe	Zn	Cu	Mn	Ni	B	Place country	Studied species (Sp.)
Current study (untreated)	30.32–62.04	28.11–67.60	3.62–4.69	0.96–2.09	2.88–3.27	28.03–44.57	Aqaba Gulf, Egypt	3 Sp. (*Siganus luridus, Lethrinus borbonicus,* and *Cheilinus lunulatus*)
Ismail and Abu-Hilal^37^*	1.77–2.60	2.21–4.45	0.18–0.26	0.19–0.22	0.40–0.53	–	Aqaba Gulf, Jordan	3 Sp. (*Ctenochaetus striatus, Zebrasoma xanthurum,* and *Scarus ferrugineus*)
El-Moselhy et al.^38^	1.15–10.9	2.70–8.23	0.170–0.740	0.10–0.93	–	–	Hurghada, Red Sea, Egypt	14 Sp. (*Epinephelus sp., Caranx sp., Scarus gibbus, Nemipterus japonicus, Sardinella sp., Synodus sp., Carangoides bajad, Lutjanus bohar, Thunnus albacares, Gerres oyena, Sargocentron spiniferum, Siganus rivulatus, Lethrinus sp. and Trachurus mediterraneus*)
Yüksel et al.^39^	1.56–7.61	4.39–5.85	0.10–0.198	0.31–1.16	0.009–0.06	–	Miliç Wetland, Türkiye	3 Sp. (*Esox lucius, Squalius cephalus, and Carassius gibelio*)
Mziray and Kimirei^40^	34.02–103.29	67.8–214.6	1.65–4.71	5.55–10.18	0.12–0.15	–	Dar es Salaam, Tanzania	3 Sp. (*Siganus sutor, Lethrinus harak*, and *Rastrelliger kanagurta*)
Abbas et al.^41^	36.86–135.96	11.95–35.18	10.33–25.85	1.27–2.50	1.46–4.86	2.50–8.07	Suez Gulf, Egypt	5 Sp. (*Cephalopholis hemistiktos, Lethrinus mahsena, Pagrus major, Cheilinus lunulatus, and Carangoides bajad*)
El-Shorbagy et al.^42^	31.80–81.35	7.02–19.75	2.11–10.29	0.50–1.31	1.20–1.76	–	Aqaba Gulf, Egypt	8 Sp. (*Lethrinus ramak, Cephalopholis hemistiktos, Pagellus affinis, Trachurus japonicus, Cheilinus lunulatus, Siganus luridus, Parupeneus forsskali,* and *Caesio suevica*)
Younis et al.^43^*	17.0–39.29	–	1.60–3.94	0.65–1.13	3.60–19.19	–	Red Sea, Saudi Arabia	5 Sp. (*Epinephelus chlorostigma, (Plectropomus areolatus, Parastromateus niger, Pristipomoides multidens, and Polysteganus coeruleopunctatus*)
Zaghloul et al.^44^	13.6–29.1	5.90–11.9	0.381–0.970	0.264–0.897	0.332–0.585	–	Hurghada, Red Sea, Egypt	5 Sp. (*Gerres oyena, Scarus sordidus, Lethrinus lentjan, Siganus rivulatus, and Mulloidichthys vanicolensis*)
Ben-Tahar et al.^45^	15.56–30.75	4.47–11.59	0.41–0.82	–	–	–	Betoya Bay, Morocco	3 Sp. (*Sardina pilchardus, Engraulis encrasicolus, Trachurus trachurus*)
Hossain et al.^46^*	–	5.78–9.56	0.61–3.10	–	0.004–0.17	–	Meghna River, Bangladesh	15 Sp. (*Oreochromis mossambicus, Anabas testudineus, Gibelion catla, Labeo rohita, Ctenopharyngodon idella, Aristichthys nobilis, Labeo calbasu, Cirrhinus reba, Ompok pabda, Otolothoides pama, Apocryptes bato, Polynemus paradiseus, Mystus gulio, Harpadon nehereus, and Lates calcarifer*)

*Fish dry weight (dw-b) was converted to wet weight (ww basis) using a conversion coefficient of 4.8^47^.

#### Iron Levels in fish and their soaked samples

The experiment demonstrated that the iron level in the flesh of sigan, bongos, and mallas fish were varied significantly depending on the soaking treatments. In all species, the control (soaked in water) exhibited the highest iron levels. For sigan fish, the control had 62.04 mg/kg, while the salt-soaked, vinegar soaked, and mixed soaked reduced the iron level to 22.82 mg/kg, 30.00 mg/kg, and 20.10 mg/kg, respectively. Similarly, *bongos fish* showed 39.42 mg/kg in the control, with reductions to 23.01 mg/kg, 26.06 mg/kg, and 17.46 mg/kg in salt-soaked, vinegar soaked, and mixed soaked. For *Mallas fish*, the control had 30.32 mg/kg, with the other treatments showing reductions to 22.09 mg/kg, 21.23 mg/kg, and 14.22 mg/kg. The mixed soaked consistently showed the most significant reduction in iron level across all species, indicating a synergistic effect between salt and vinegar in reducing iron levels.

In terms of Fe, sigan fish again showed the highest value of 62.04 mg/kg, with mallas fish displaying lower levels at 30.32 mg/kg, which are significantly higher than those reported by Ismail and Abu-Hilal^37^ in three fish species collected from the Aqaba Gulf, Red Sea (1.77–2.60 mg/kg), El-Moselhy et al.^38^ in 14 fish species collected from the Hurghada city, Red Sea (1.15–10.9 mg/kg), and Yüksel et al.^39^ in three fish species collected from the Miliç Wetland, Türkiye (1.56–7.61 mg/kg). In contrast, the results of the current study align more closely with the findings of Mziray and Kimirei^40^ in three fish species collected from the Dar es Salaam, Tanzania (34.02–103.29 mg/kg), Abbas et al.^41^ in five fish species collected from the Suez Gulf, Red Sea (36.86–135.96 mg/kg), and El-Shorbagy et al.^42^ in eight fish species collected from the Aqaba Gulf, Red Sea (31.80–81.35 mg/kg). Additionally, the iron levels in the current study were relatively higher than those reported by Younis et al.^43^ in five fish species collected from the Red Sea, Saudi Arabia (17.0–39.29 mg/kg). On the other hand, the values recorded in Zaghloul et al.^44^ in five fish species collected from the Hurghada city, Red Sea (13.6–29.1 mg/kg) and Ben-Tahar et al.^45^ in three fish species collected from the Betoya Bay, Morocco (15.56–30.75 mg/kg) were lower than those of the current study.

#### B levels in fish and their soaked samples

The level of meal B in the flesh of sigan, bongos, and mallas fish varied significantly with different soaking treatments. For sigan fish, the control sample had a level of 28.03 mg/kg, while the salt-soaked, vinegar soaked, and mixed soaked treatments resulted in levels of 21.41 mg/kg, 32.50 mg/kg, and 21.78 mg/kg, respectively. In bongos fish, the control exhibited 32.21 mg/kg, and the salted soaked caused an increase to 38.36 mg/kg. However, the vinegar soaked and mixed soaked resulted in a significant decrease to 8.76 mg/kg and 5.87 mg/kg, respectively. For mallas fish, the control showed 44.57 mg/kg, while the salt treatment (salt-soaked) caused a reduction to 18.81 mg/kg, with the vinegar soaked and mixed soaked treatments leading to levels of 30.33 mg/kg and 20.32 mg/kg, respectively. These findings higher than that reported by Abbas et al.^41^ whom reported that the levels of boron in the muscles of in five fish species collected from the Suez Gulf, Red Sea were fluctuated between 2.50 ± 0.69 mg/kg in *Cephalopholis hemistiktos* and 8.07 ± 0.37 mg/kg in *Carangoides bajad.* Also, it was higher than those reported by Ekraim et al.^48^ in *Siganus rivulatus* (sigan fish) collected from the Suez Gulf, Red Sea (8.32 ± 0.53 mg/kg). However, the B levels observed in the present study are within range to those recorded by Abbas and Alnasser^49^ in two coral reef fish collected from the Suez Gulf, Red Sea (33.20–45.95 mg/kg), and Abbas et al.^50^ in three fish species collected from the Suez Gulf, Red Sea (28.90 ± 1.5 2–45.95 ± 2.4 8 mg/kg),

#### Cu levels in fish and their soaked samples

Cu plays vital roles in iron utilization and acts as a cofactor for enzymes essential in glucose metabolism, as well as in the synthesis of haemoglobin, connective tissue, and phospholipids^51,52^. However, Cu, can cause acute poisoning if consumed in excess levels. Consequently, regulatory bodies have established safe Cu levels, due to the fact that Cu may bioaccumulate and pose serious threats when their levels exceed permissible levels^53–55^. Although Cu from marine fish is not considered carcinogenic to humans or animals, it is likely to cause liver and kidney damage if consumed in excess levels^56,57^. The level of Cu in the fish fillets varied significantly depending on the soaking treatment. For *Sigan fish*, the control showed a level of 4.69 mg/kg, while the salted soaked resulted in a decrease to 2.90 mg/kg. The vinegar soaked caused an increase to 5.75 mg/kg, and the mixed soaked reduced the level to 3.85 mg/kg. In *Bongos fish*, the control recorded 3.84 mg/kg, with the salted soaked increasing it to 4.74 mg/kg, while the vinegar soaked and mixed soaked treatments resulted in levels of 4.14 mg/kg and 2.78 mg/kg, respectively. For mallas fish, the control exhibited 3.62 mg/kg, while the salted soaked led to a slight increase to 4.86 mg/kg. The vinegar soaked showed 4.55 mg/kg, and the mixed soaked lowered the level to 3.05 mg/kg. These results suggest that the effects of the treatments on Cu levels were species-specific, with the apple vinegar treatment generally increasing the Cu level, while the mixed soaked had a reducing effect in some species.

For Cu, sigan fish exhibited the highest level at 4.69 mg/kg, and *Mallas fish* at the lowest level of 3.62 mg/kg, which are higher than those reported by several earlier studies. For instance, Ismail and Abu-Hilal^37^ in three fish species collected from the Aqaba Gulf, Red Sea (0.18–0.26 mg/kg), El-Moselhy et al.^38^ in 14 fish species collected from the Hurghada city, Red Sea (0.170–0.740 mg/kg), Yüksel et al.^39^ in three fish species collected from the Miliç Wetland, Türkiye (0.10–0.198 mg/kg), and Ben-Tahar et al.^45^ in three fish species collected from the Betoya Bay, Morocco (0.41–0.82 mg/kg) reported lower Cu levels compared to the current study region. The Cu levels observed in the present study are comparable to those recorded by Mziray and Kimirei^40^ in three fish species collected from the Dar es Salaam, Tanzania (1.65–4.71 mg/kg) and Younis et al.^43^ in five fish species collected from the Red Sea, Saudi Arabia (1.60–3.94 mg/kg). On the other hand, higher Cu levels were reported by Abbas et al.^41^ in five fish species collected from the Suez Gulf, Red Sea (10.33–25.85 mg/kg), El-Shorbagy et al.^42^ in eight fish species collected from the Aqaba Gulf, Red Sea (2.11–10.29 mg/kg) and Hossain et al.^46^ in 15 fish species collected from the Meghna River, Bangladesh (0.61–3.10 mg/kg).

#### Mn levels in fish and their soaked samples

The level of Mn in the fish fillets varied depending on the soaking treatment. For *Sigan fish*, the control exhibited a level of 2.09 mg/kg, which significantly decreased in the salted soaked to 0.97 mg/kg. The vinegar soaked resulted in 1.50 mg/kg, while the mixed soaked produced 1.01 mg/kg. In bongos fish, the control had 0.96 mg/kg, with the salted soaked leading to an increase to 1.18 mg/kg. The vinegar soaked showed 0.98 mg/kg, and the mixed soaked resulted in a decrease to 0.66 mg/kg. For *Mallas fish*, the control recorded 1.22 mg/kg, and the salted soaked caused a slight decrease to 0.97 mg/kg. The vinegar soaked showed 1.01 mg/kg, and the mixed soaked led to the lowest level of 0.68 mg/kg. These results suggest that the effects of the treatments on Mn levels were species-specific, with the Mixed soaked generally leading to the lowest levels of Mn in all species.

Mn levels were also highest in sigan fish at 2.09 mg/kg, with bongos fish showing lower values of 0.96 mg/kg, which were higher than those reported in several earlier studies. For example, Ismail and Abu-Hilal^37^ in three fish species collected from the Aqaba Gulf, Red Sea (0.19–0.22 mg/kg), El-Moselhy et al.^38^ in 14 fish species collected from the Hurghada city, Red Sea (0.10–0.93 mg/kg), Zaghloul et al.^44^ in five fish species collected from the Hurghada city, Red Sea (0.264–0.897 mg/kg), and Yüksel et al.^39^ in three fish species collected from the Miliç Wetland, Türkiye (0.31–1.16 mg/kg) observed lower Mn levels in fish samples from their respective studies. The Mn levels recorded in this study align closely with those reported by Younis et al.^43^ in five fish species collected from the Red Sea, Saudi Arabia (0.65–1.13 mg/kg) and El-Shorbagy et al.^42^ in eight fish species collected from the Aqaba Gulf, Red Sea (0.50–1.31 mg/kg). However, significantly higher Mn levels were documented by Mziray and Kimirei^40^ in three fish species collected from the Dar es Salaam, Tanzania (5.55–10.18 mg/kg) and Abbas et al.^41^ in five fish species collected from the Suez Gulf, Red Sea (1.27–2.50 mg/kg).

#### Ni levels in fish and their soaked samples

The level of Ni in the fish fillets varied significantly depending on the soaking treatment. In sigan fish, the control sample exhibited the highest Ni level of 3.27 mg/kg, which decreased to 1.95 mg/kg in the salted soaked. The vinegar soaked resulted in 3.00 mg/kg, while the mixed soaked produced 2.01 mg/kg. In *Bongos fish*, the control had 2.88 mg/kg, and the salted soaked led to a slight decrease to 2.38 mg/kg. The vinegar soaked increased the level to 3.46 mg/kg, and the mixed soaked reduced it to 2.32 mg/kg. For mallas fish, the control showed 2.91 mg/kg, which decreased to 2.19 mg/kg with the salt treatment (Salt-Soaked). The vinegar soaked resulted in 3.03 mg/kg, and the mixed soaked reduced it to 2.03 mg/kg. These results suggest that the effects of the treatments on Ni levels were species-specific, with the Mixed soaked generally leading to the lowest levels of Ni.

Ni was found to be most concentrated in sigan fish at 3.27 mg/kg, and *Bongos fish* with the lowest level of 2.88 mg/kg, which were notably higher than those reported in several prior studies. For instance, Ismail and Abu-Hilal^37^ in three fish species collected from the Aqaba Gulf, Red Sea (0.40–0.53 mg/kg), Mziray and Kimirei^40^ in three fish species collected from the Dar es Salaam, Tanzania (0.12–0.15 mg/kg), Zaghloul et al.^44^ in five fish species collected from the Hurghada city, Red Sea (0.332–0.585 mg/kg), and El-Shorbagy et al.^42^ in eight fish species collected from the Aqaba Gulf, Red Sea (1.20–1.76 mg/kg) all documented lower Ni levels in fish samples. In contrast, the current results are within the range reported by Abbas et al.^41^ in five fish species collected from the Suez Gulf, Red Sea (1.46–4.86 mg/kg) and are lower than the maximum values recorded by Younis et al.^43^ in five fish species collected from the Red Sea, Saudi Arabia (3.60–19.19 mg/kg). The findings of this study are significantly higher than the levels reported by Hossain et al.^46^ in 15 fish species collected from the Meghna River, Bangladesh (0.004–0.17 mg/kg) and Yüksel et al.^39^ in three fish species collected from the Miliç Wetland, Türkiye (0.009–0.06 mg/kg), suggesting elevated Ni pollution in the current study area.

#### Zn levels in fish and their soaked samples

Zn is an essential meal for aquatic organisms, particularly fish. However, high Zn levels in the environment can be harmful, because they interfere with the metabolism and uptake of calcium. Fish acquire Zn from both water and their food^58^. Zn serves as a cofactor for approximately 300 enzymes, aiding in key activities including RNA and DNA metabolism. It also crucial for the stability of several proteins, including signalling enzymes involved in cellular signal transduction^59^. Nonetheless, prolonged excessive intake of Zn may cause iron and Cu deficits, in addition to symptoms including nausea, vomiting, fatigue, fever, headache, exhaustion, and abdominal pains^60^. The level of Zn in the fish fillets showed significant variation based on the soaking treatments. In sigan fish, the control exhibited the highest level of 67.60 mg/kg, which decreased sharply to 14.69 mg/kg with the salted soaked. The vinegar soaked reduced the level to 35.25 mg/kg, and the mixed soaked led to 23.62 mg/kg. In *Bongos fish*, the control sample showed 44.58 mg/kg, which decreased to 18.70 mg/kg in the salt treatment (Salt-Soaked). The vinegar soaked resulted in 15.67 mg/kg, while the mixed soaked exhibited the lowest level of 10.50 mg/kg. For Mallas fish, the control level was 28.11 mg/kg, which decreased to 16.04 mg/kg in the salted soaked. The vinegar soaked reduced it further to 14.66 mg/kg, while the mixed soaked showed the lowest level of 9.82 mg/kg. These results suggest that all treatments, especially the mixed soaked, significantly reduced the Zn level in the fish fillets across all species.

Zn levels were highest in sigan fish (67.60 mg/kg), and *Mallas fish* with the lowest level at 28.11 mg/kg, which are significantly higher than those reported by Ismail and Abu-Hilal^37^ in three fish species collected from the Aqaba Gulf, Red Sea (2.21–4.45 mg/kg), El-Moselhy et al.^38^ in 14 fish species collected from the Hurghada city, Red Sea (2.70–8.23 mg/kg), Hossain et al.^46^ in 15 fish species collected from the Meghna River, Bangladesh (5.78–9.56 mg/kg), and Yüksel et al.^39^ in three fish species collected from the Miliç Wetland, Türkiye (4.39–5.85 mg/kg). The Zn levels in the current study are closer to the values reported by Abbas et al.^41^ in five fish species collected from the Suez Gulf, Red Sea (11.95–35.18 mg/kg) and El-Shorbagy et al.^42^ in eight fish species collected from the Aqaba Gulf, Red Sea (7.02–19.75 mg/kg), although the upper range in the present study exceeds those studies. The levels observed by Zaghloul et al.^44^ in five fish species collected from the Hurghada city, Red Sea (5.90–11.9 mg/kg) and Ben-Tahar et al.^45^ in three fish species collected from the Betoya Bay, Morocco (4.47–11.59 mg/kg) were also lower, further highlighting the higher Zn levels in the current study. Interestingly, the results from Mziray and Kimirei^40^ in three fish species collected from the Dar es Salaam, Tanzania (67.8–214.6 mg/kg) show significantly higher Zn levels than those recorded in the present study.

In general, the inorganic substances in the soaking solution may have moved into the fish tissue, as indicated by the increase in certain HMC levels following soaking treatments^61^. On the other hand, the dropping of metal contents during soaking, salting, cooking, and smoking, however, may be the result of these metals dripping off after processing as free salts or possibly combining with bound metal proteins, soluble amino acids, or the dropped pH in acidic surroundings^61–63^.

### Pearson correlation coefficient

**Fig. 3:**
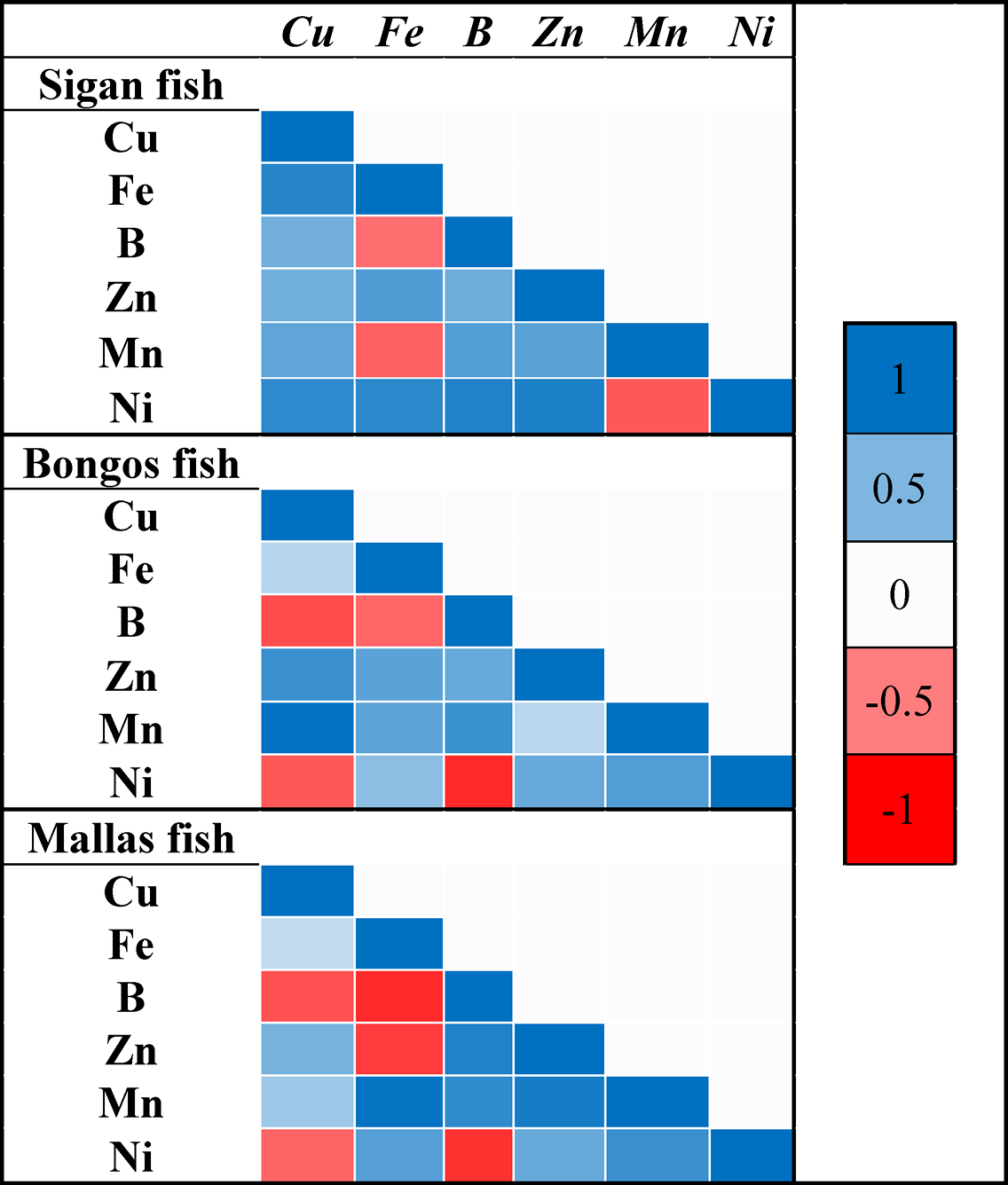
Heatmap showing Pearson correlation coefficients based on HMCs in three marine fish fillets from Newaba City, Aqaba Gulf, Red Sea.

Figure 3 illustrates the Pearson correlation (r) values, revealing interrelationships between HMCs in the muscle tissues of three marine fish species from Nuweiba City, Aqaba Gulf, Red Sea. Positive correlations suggest shared sources or environmental factors, while negative correlations indicate distinct accumulation mechanisms. In sigan fish, Cu strongly correlates with Fe (0.85) and Ni (0.82), reflecting shared bioaccumulation pathways. Iron also shows positive correlations with Ni (0.83) and Zn (0.69) but negatively correlates with barium (B) (-0.56). Barium correlates positively with Zn (0.57) and Mn (0.68), while Mn is negatively correlated with Ni (-0.65) but positively with barium (0.68), Zn (0.64), and Cu (0.62).

In bongos fish, Cu exhibits a strong correlation with Mn (0.99) and moderate correlations with Zn (0.77). Iron shows a weaker correlation with Cu (0.27) but stronger with Zn (0.64). Barium negatively correlates with Ni (-0.84), indicating distinct sources. Ni shows weaker positive correlations with Zn (0.59) and Mn (0.68).

In mallas fish, Cu shows weak positive correlations with iron (0.24) and moderate with Zn (0.53), while barium negatively correlates with Cu (-0.67) and iron (-0.83). Zn strongly correlates with barium (0.88) and Mn (0.92). Mn also correlates positively with iron (0.98), while Ni negatively correlates with Cu (-0.60) and barium (-0.80) but moderately with Zn (0.59) and Mn (0.77). Negative correlations suggest the presence of distinct sources, potentially involving different chemicals or dyes^64^. However, positive significant correlation suggests that the parameters have a relationship with each other and could have developed from similar sources and similar behaviors^65,66^. Comparatively, Zn, Mn, and barium consistently exhibit positive correlations across species, suggesting shared accumulation dynamics. However, Cu, iron, and Ni show species-specific variations. These findings highlight the complex, species-specific dynamics of HMCs accumulation in marine environments, emphasizing the need for targeted pollution monitoring to protect ecological and human health.

### Environmental pollution indicators

The degree of contamination was evaluated by the contamination factor and the meal pollution index^67^.

#### Pollution index evaluation

**Fig. 4:**
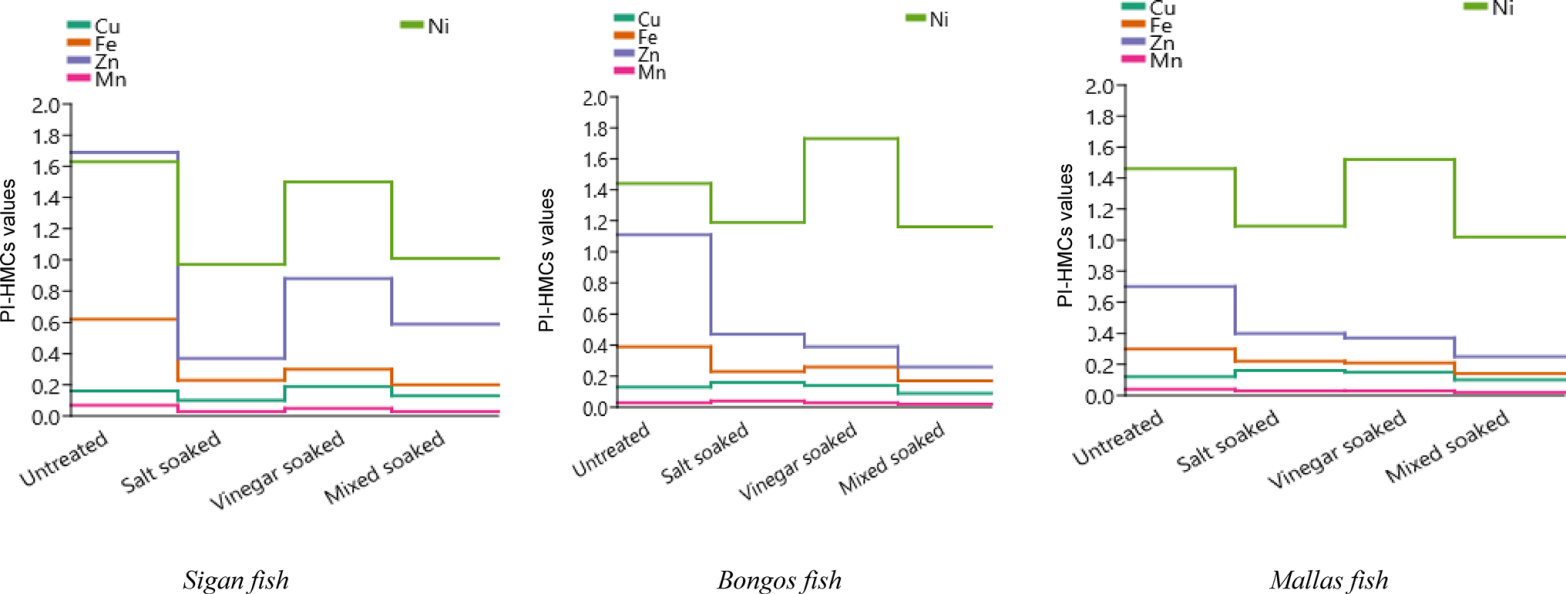
Impact of soaking treatments on pollution index (PI-HMCs) of HMCs in three marine fish from Newaba City, Aqaba Gulf, Red Sea.

The pollution index (PI-HMCs) of HMCs in the three fish species were assessed under various soaking treatments (salt-soaked, vinegar soaked, and mixed soaked) compared to untreated control samples (C) were represented in Fig. 4. The results demonstrated varying degrees of contamination and the effectiveness of soaking in reducing HMCs, underscoring its potential in improving food safety and environmental protection.

The PI-HMCs values offer a clear indication of the contamination level. PI-HMCs < 0.2 indicates no significant contamination, 0.2 < PI-HMCs < 0.6 represents minor contamination, 0.6 < PI-HMCs < 1 suggests a moderate contamination, and PI-HMCs > 1 indicates a severe contamination and the residual metal i in fish being out of threshold values^68^.

For *Sigan fish*, Cu and Mn exhibited minimal contamination (CL < 1), indicating negligible environmental impact. Fe showed significant reductions, decreasing from 0.62 in the control to 0.20 in mixed soaked, all within the negligible contamination category. Zn exhibited low contamination, with the control group slightly exceeding the threshold (1.69), but soaking treatments effectively reduced levels below 1. Ni presented the highest contamination levels among the metals tested, with the control showing low contamination (1.63), and soaking treatments reducing values to the range of 0.97–1.50, still within the low contamination category. For *bongos fish*, Cu levels were consistently minimal across all groups, ranging from 0.09 to 0.16, indicating very low contamination. Fe levels decreased significantly from 0.39 in the control to 0.17 in mixed soaked, remaining in the negligible contamination range. Zn showed low contamination in the control (1.11), but soaking treatments further reduced levels to below the low contamination threshold, with values ranging from 0.26 to 0.47. Mn levels remained minimal (CL < 1) across all treatments. Ni, the most prominent contaminant, exhibited low contamination levels, with values ranging from 1.16 to 1.73, suggesting the need for further attention to its reduction.

For *mallas fish*, Cu levels were also minimal, ranging from 0.10 to 0.16, all below the contamination threshold. Fe decreased markedly from 0.30 in the control to 0.14 in mixed soaked, classified as negligible contamination. Zn levels declined from 0.70 in the control to 0.25 in mixed soaked, reflecting a significant reduction. Mn levels were consistently low (0.02–0.04). Ni exhibited the highest contamination levels, with the control (1.46) and soaking treatments (1.09–1.52) remaining within the low contamination category. Overall, the results highlight the efficacy of soaking treatments in significantly reducing contamination levels of Cu, Fe, Zn, and Mn in all three species and mitigating Ni levels to a lesser extent. These findings emphasize the importance of such treatments as an intervention to improve the safety and quality of marine fish products, aligning with public health objectives and environmental conservation efforts.

#### Metal pollution index (MPI-HMCs)

**Fig. 5:**
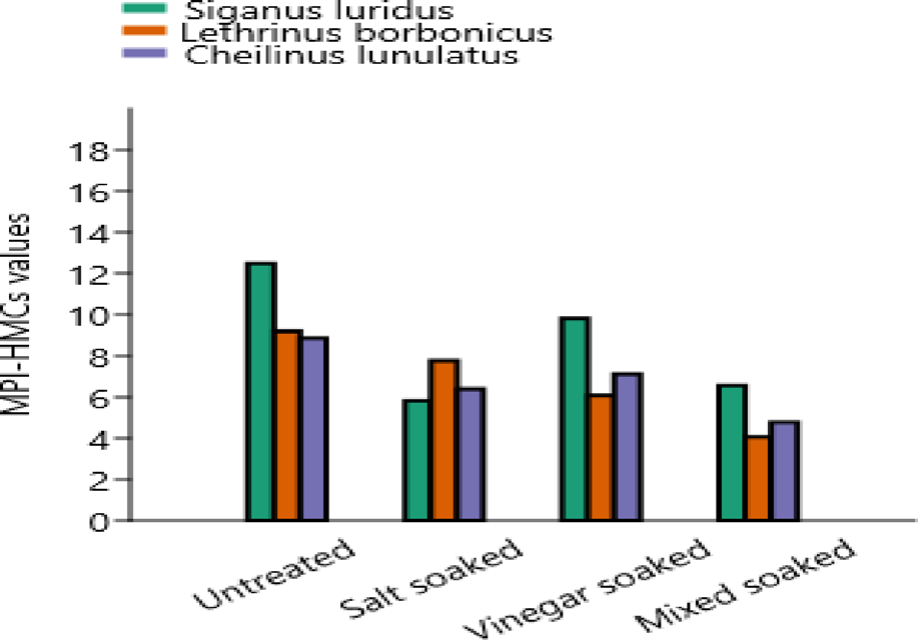
Impact of soaking treatments on MPI-HMCs values in three marine fish fillets from Newaba City, Aqaba Gulf, Red Sea.

Metal pollution index (MPI-HMCs) is a tool used to assess the overall pollution level in fish based on the HMCs^29^. The interpretation of MPI-HMCs values is as follows: MPI-HMCs ≤ 1 is considered safe, 2 < MPI-HMCs ≤ 3 indicates slightly polluted, 3 < MPI-HMCs ≤ 5 signifies moderately polluted, and MPI-HMCs > 10 is categorized as heavily polluted^28^. Based on the provided data (Fig. 5), we can assess the contamination levels of *sigan*, *bongos*, and *mallas fish* under various soaking treatments (salt-soaked, vinegar soaked, mixed soaked) in comparison to the untreated control group. For *sigan fish*, the control group showed the highest contamination level at 12.48, with soaking treatments reducing the contamination in all cases. The salt-soaked treatment resulted in a reduction to 5.83, while vinegar soaked and mixed soaked also showed improvements, with contamination levels of 9.81 and 6.57, respectively. Although the soaking treatments helped reduce the contamination levels, they did not bring them to a significantly low level. The overall reduction indicates that soaking treatments can help mitigate the contamination, but the levels remain relatively high compared to the other species. In bongos fish, the control group had a contamination level of 9.19, which decreased across all treatments. mixed soaked was the most effective soaking treatment, with a contamination level of 4.07, showing the most significant reduction. Salt-soaked and vinegar soaked also showed reductions, with contamination levels of 7.77 and 6.08, respectively. This suggests that soaking treatments can effectively reduce contamination levels in bongos fish, with mixed soaked providing the best results. For mallas fish, the control group exhibited a contamination level of 8.87, which also decreased with soaking treatments. Mixed soaked again showed the most significant reduction, with a contamination level of 4.78, while salt-soaked and vinegar soaked showed less dramatic reductions, with contamination levels of 6.40 and 7.13, respectively. Soaking treatments were effective in reducing contamination, with mixed soaked being the most effective. In conclusion, the soaking treatments generally helped reduce contamination levels across all three fish species. While sigan fish showed a moderate reduction, bongos and mallas fish exhibited more significant reductions, especially with the mixed soaked treatment. These results suggest that soaking treatments can be an effective method for reducing contamination levels in marine fish, with varying effectiveness depending on the species and the treatment used. The low levels of MPI-HMCs in muscle might be related to low metabolic activity and low metal-binding proteins in muscles^69,70^. The MPI-HMCs value of *Argyrosomus regius* 0.1^71^, sardines and anchovies 0.46–0.76 and 0.65–0.89, respectively^72^, demersal and pelagic fish 3.65–4.70, respectively^73^.

### Assessing human health risks of HMCs exposure

Human health risk assessment has been extensively employed to evaluate the potential risk associated with the contaminated-fish consumption, and provide evidence of risk to the decision-makers^74^. To evaluate the human health risks, EDI-HMCs and HQ-HMCs, and HI-HMCs methods were used. Fish serve as a major dietary protein source globally. Yet they are also recognized as significant bio-accumulators of metals due to their apex position in the nutritional web and food chain within both freshwater^75^ and marine environments^76^. The potential hazards to human well-being and environmental integrity can arise from excessive levels of metals, which include transition metals and metalloid metals. To effectively select a species as a bio-indicator for assessing human health risks, it must satisfy specific criteria: it should be commonly consumed in the region, exhibit wide geographic distribution, be able to accumulate substantial levels of metals, and have sufficient tissue mass to facilitate residue analysis^77^.

#### EDI-HMCs values

The estimated daily intake (EDI-HMCs) values of HMCs were determined for three fish species under various soaking treatments. The soaking treatments included different solutions such as salt and vinegar -infused solutions. The values of EDI-HMCs for Cu, Fe, Mn, Ni, and Zn were significantly lower than the acceptable daily intake (ADI) for each metal. For example, the Cu ADI is 50 mg/kg, iron ADI is 70 mg/kg, Mn ADI is 1.4 mg/kg, Ni ADI is 0.04 mg/kg, and Zn ADI is 50 mg/kg^30^

The EDI-HMCs values of Cu for the three studied fish species varied across different soaking treatments (Table 2), yet remained well below the acceptable threshold set by the ATDI (50 mg/kg body weight/day). For sigan fish, the EDI-HMCs ranged from 1.20E−02 to 2.50E−02 mg/kg/day for children, 4.50E−03 to 8.90E−03 mg/kg/day for young individuals, and 2.60E−03 to 5.10E−03 mg/kg/day for adults. In the case of *Bongos fish*, the EDI-HMCs values spanned 1.20E−02 to 2.00E−02 mg/kg/day for children, 4.30E−03 to 7.40E−03 mg/kg/day for young individuals, and 2.50E−03 to 4.20E−03 mg/kg/day for adults. Similarly, for *mallas fish*, the EDI-HMCs ranged between 1.30E−02 and 2.10E−02 mg/kg/day for children, 4.70E−03 to 7.60E−03 mg/kg/day for young individuals, and 2.70E−03 to 4.30E−03 mg/kg/day for adults. Across all species and treatments, children consistently exhibited the highest EDI-HMCs values, while young and adult consumers had significantly lower exposure.

**Table 2:** Impact of soaking treatments on estimated daily intake (EDI-HMCs) of HMCs in three marine fish fillets for children, young, and adults consumers: variation based on different soaking treatments.

	Sigan fish			Bongos fish			Mallas fish		
	Children	Young	Adults	Children	Young	Adults	Children	Young	Adults
EDI-Cu									
Untreated	2.0E−02	7.3E−03	4.2E−03	1.6E−02	6.0E−03	3.4E−03	1.5E−02	5.6E−03	3.2E−03
Salt-soaked	1.2E−02	4.5E−03	2.6E−03	2.0E−02	7.4E−03	4.2E−03	2.1E−02	7.6E−03	4.3E−03
Vinegar soaked	2.5E−02	8.9E−03	5.1E−03	1.8E−02	6.4E−03	3.7E−03	1.9E−02	7.1E−03	4.0E−03
Mixed soaked	1.6E−02	6.0E−03	3.4E−03	1.2E−02	4.3E−03	2.5E−03	1.3E−02	4.7E−03	2.7E−03
EDI-Fe									
Untreated	2.6E−01	9.7E−02	5.5E−02	1.7E−01	6.1E−02	3.5E−02	1.3E−01	4.7E−02	2.7E−02
Salt-soaked	9.7E−02	3.6E−02	2.0E−02	9.8E−02	3.6E−02	2.0E−02	9.4E−02	3.4E−02	2.0E−02
Vinegar soaked	1.3E−01	4.7E−02	2.7E−02	1.1E−01	4.1E−02	2.3E−02	9.1E−02	3.3E−02	1.9E−02
Mixed soaked	8.6E−02	3.1E−02	1.8E−02	7.4E−02	2.7E−02	1.6E−02	6.1E−02	2.2E−02	1.3E−02
EDI-B									
Untreated	1.2E−01	4.4E−02	2.5E−02	1.4E−01	5.0E−02	2.9E−02	1.9E−01	6.9E−02	4.0E−02
Salt-soaked	9.1E−02	3.3E−02	1.9E−02	1.6E−01	6.0E−02	3.4E−02	8.0E−02	2.9E−02	1.7E−02
Vinegar soaked	1.4E−01	5.1E−02	2.9E−02	3.7E−02	1.4E−02	7.8E−03	1.3E−01	4.7E−02	2.7E−02
Mixed soaked	9.3E−02	3.4E−02	1.9E−02	2.5E−02	9.1E−03	5.2E−03	8.7E−02	3.2E−02	1.8E−02
EDI-Zn									
Untreated	2.9E−01	1.1E−01	6.0E−02	1.9E−01	6.9E−02	4.0E−02	1.2E−01	4.4E−02	2.5E−02
Salt-soaked	6.3E−02	2.3E−02	1.3E−02	8.0E−02	2.9E−02	1.7E−02	6.8E−02	2.5E−02	1.4E−02
Vinegar soaked	1.5E−01	5.5E−02	3.1E−02	6.7E−02	2.4E−02	1.4E−02	6.3E−02	2.3E−02	1.3E−02
Mixed soaked	1.0E−01	3.7E−02	2.1E−02	4.5E−02	1.6E−02	9.3E−03	4.2E−02	1.5E−02	8.7E−03
EDI-Mn									
Untreated	8.9E−03	3.3E−03	1.9E−03	4.1E−03	1.5E−03	8.5E−04	5.2E−03	1.9E−03	1.1E−03
Salt-soaked	4.2E−03	1.5E−03	8.7E−04	5.1E−03	1.8E−03	1.1E−03	4.1E−03	1.5E−03	8.6E−04
Vinegar soaked	6.4E−03	2.3E−03	1.3E−03	4.2E−03	1.5E−03	8.7E−04	4.3E−03	1.6E−03	9.0E−04
Mixed soaked	4.3E−03	1.6E−03	8.9E−04	2.8E−03	1.0E−03	5.9E−04	2.9E−03	1.1E−03	6.0E−04
EDI-Ni									
Untreated	1.4E−02	5.1E−03	2.9E−03	1.2E−02	4.5E−03	2.6E−03	1.2E−02	4.5E−03	2.6E−03
Salt-soaked	8.3E−03	3.0E−03	1.7E−03	1.0E−02	3.7E−03	2.1E−03	9.3E−03	3.4E−03	1.9E−03
Vinegar soaked	1.3E−02	4.7E−03	2.7E−03	1.5E−02	5.4E−03	3.1E−03	1.3E−02	4.7E−03	2.7E−03
Mixed soaked	8.6E−03	3.1E−03	1.8E−03	9.9E−03	3.6E−03	2.1E−03	8.7E−03	3.2E−03	1.8E−03

The estimated daily intake (EDI-HMCs) values for Fe across the three fish species and various treatments were analyzed and compared with the acceptable daily intake threshold (ATDI) of 70 mg/kg body weight/day. For sigan fish, the EDI-HMCs values ranged from 8.60E−02 to 2.60E−01 mg/kg/day for children, 3.10E−02 to 9.70E−02 mg/kg/day for young individuals, and 1.80E−02 to 5.50E−02 mg/kg/day for adults. The highest values were observed under control conditions (C), while treatment mixed soaked, involving soaking in a salt and vinegar solution, resulted in the lowest EDI-HMCs values for all consumer groups, significantly reducing iron exposure. In *Bongos fish*, the EDI-HMCs values varied between 7.40E−02 and 1.70E−01 mg/kg/day for children, 2.70E−02 to 6.10E−02 mg/kg/day for young individuals, and 1.60E−02 to 3.50E−02 mg/kg/day for adults. Treatment SALT-SOAKED, involving a salt solution soak, showed a notable reduction in EDI-HMCs values compared to the control, with MIXED SOAKED further lowering the intake for all age groups. For *Mallas fish*, the EDI-HMCs ranged from 6.10E−02 to 1.30E−01 mg/kg/day for children, 2.20E−02 to 4.70E−02 mg/kg/day for young individuals, and 1.30E−02 to 2.70E−02 mg/kg/day for adults. As with the other species, the control group (C) displayed the highest EDI-HMCs values, while mixed soaked yielded the lowest values. Across all treatments and fish species, the EDI-HMCs values for iron were substantially below the ATDI of 70 mg/kg/day, ensuring safe consumption levels. Children consistently exhibited the highest EDI-HMCs values across all groups, followed by young individuals and adults. Soaking treatments, particularly mixed soaked, effectively minimized iron exposure, highlighting the benefits of preprocessing fish prior to consumption.

The estimated daily intake (EDI-HMCs) values for B across three fish species under various treatments (C, salt-soaked, vinegar soaked, mixed soaked) were analyzed and compared across children, young individuals, and adults. The results indicated that children were the most exposed group, with EDI-HMCs values ranging from 1.20E−01 to 1.90E−01 mg/kg/day for sigan fish, from 1.40E−01 to 1.60E−01 mg/kg/day for bongos fish, and from 1.90E−01 to 1.30E−01 mg/kg/day for mallas fish. Overall, the highest EDI-HMCs values were observed in the control group (C), while treatment MIXED SOAKED, which involved a specific processing method, proved to be the most effective in reducing B exposure across all age groups, leading to a significant decrease in EDI-HMCs values for all species. Although the recorded values were well below the acceptable daily intake limits for B, these findings emphasize the importance of preprocessing fish to reduce exposure, especially in children, who exhibited higher levels of intake compared to young individuals and adults.

The estimated daily intake (EDI-HMCs) values for Zn across three fish species under various treatments (C, salt-soaked, vinegar soaked, mixed soaked) were analyzed and compared across different age groups (children, young individuals, and adults), with the acceptable daily intake (ATDI) threshold for Zn being 50 mg/kg body weight/day. The results indicated that children had the highest exposure, with EDI-HMCs values ranging from 2.90E−01 to 1.20E−01 mg/kg/day for *Sigan fish*, from 1.90E−01 to 6.70E−02 mg/kg/day for *Bongos fish*, and from 1.20E−01 to 6.30E−02 mg/kg/day for *Mallas fish*. Young individuals showed lower EDI-HMCs values, ranging from 1.10E−01 to 4.40E−02 mg/kg/day for *sigan fish*, from 6.90E−02 to 2.40E−02 mg/kg/day for *bongos fish*, and from 4.40E−02 to 2.30E−02 mg/kg/day for *Mallas fish*. Adults exhibited the lowest intake, with EDI-HMCs values ranging from 6.00E−02 to 2.50E−02 mg/kg/day for sigan fish, from 4.00E−02 to 1.40E−02 mg/kg/day for *bongos fish*, and from 2.50E−02 to 1.30E−02 mg/kg/day for mallas fish. The control group (C) displayed the highest EDI-HMCs values across all species and age groups, while treatments such as salt-soaked, vinegar soaked, and especially mixed soaked significantly reduced Zn exposure. Despite these variations, all recorded EDI-HMCs values were substantially below the ATDI of 50 mg/kg/day, ensuring safe consumption levels for all age groups.

The estimated daily intake (EDI-HMCs) values for Mn across three fish species under various treatments (C, salt-soaked, vinegar soaked, mixed soaked) were analyzed and compared across different age groups (children, young individuals, and adults), with the acceptable daily intake (ATDI) threshold for Mn being 1.4 mg/kg body weight/day. The results revealed significant differences in Mn exposure between the age groups. Children exhibited the highest exposure, with EDI-HMCs values ranging from 8.90E−03 to 4.30E−03 mg/kg/day for *sigan fish*, from 4.10E−03 to 2.80E−03 mg/kg/day for bongos fish, and from 5.20E−03 to 2.90E−03 mg/kg/day for *mallas fish*. Young individuals showed moderate exposure, with EDI-HMCs values ranging from 3.30E−03 to 1.60E−03 mg/kg/day for *sigan fish*, from 1.50E−03 to 1.00E−03 mg/kg/day for bongos fish, and from 1.90E−03 to 1.10E−03 mg/kg/day for mallas fish. Adults had the lowest exposure, with EDI-HMCs values ranging from 1.90E−03 to 8.90E−04 mg/kg/day for *sigan fish*, from 8.50E−04 to 5.90E−04 mg/kg/day for bongos fish, and from 1.10E−03 to 6.00E−04 mg/kg/day for *mallas fish*. Across all species and treatments, children consistently showed the highest EDI-HMCs values, followed by young individuals, with adults having the lowest values. The control group (C) resulted in the highest EDI-HMCs values for all species and age groups, while the treatments (Salt-soaked, vinegar soaked, and mixed soaked) effectively reduced Mn exposure. All recorded EDI-HMCs values remained well below the ATDI threshold of 1.4 mg/kg/day, indicating safe consumption levels for all age groups.

The estimated daily intake (EDI-HMCs) values for Ni across three fish species under various treatments (C, salt-soaked, vinegar soaked, mixed soaked) were analyzed and compared across different age groups (children, young individuals, and adults), with the acceptable daily intake (ATDI) threshold for Ni being 0.04 mg/kg body weight/day. The results revealed that children consistently had the highest EDI-HMCs values for Ni across all species and treatments. For *sigan fish*, the EDI-HMCs ranged from 1.40E−02 to 8.30E−03 mg/kg/day for children, from 5.10E−03 to 3.00E−03 mg/kg/day for young individuals, and from 2.90E−03 to 1.70E−03 mg/kg/day for adults. In Bongos fish, children had EDI-HMCs values ranging from 1.20E−02 to 1.00E−02 mg/kg/day, young individuals had values from 4.50E−03 to 3.70E−03 mg/kg/day, and adults had values from 2.60E−03 to 2.10E−03 mg/kg/day. Similarly, for *mallas fish*, children showed EDI-HMCs values ranging from 1.20E−02 to 9.30E−03 mg/kg/day, young individuals had values from 4.50E−03 to 3.40E−03 mg/kg/day, and adults had values from 2.60E−03 to 1.90E−03 mg/kg/day. In all cases, the control group (C) exhibited the highest EDI-HMCs values, with the treatments (salt-soaked, vinegar soaked, mixed soaked) leading to a reduction in Ni intake across all age groups. Despite the variation in EDI-HMCs values between treatments and species, all values remained well below the ATDI threshold of 0.04 mg/kg/day, indicating that Ni exposure from these fish species, even at the highest EDI-HMCs values, is within safe consumption limits for all age groups.

These findings of this study suggest that the estimated daily intake of HMCs from the consumption of the studied fish species is well within the safe limits. Despite children having a higher relative exposure to these metals, the EDI-HMCs values remained significantly below the maximum allowable intake, indicating that consuming these fish poses no significant health risk when treated properly. This finding supports the conclusion that HMCs contamination in fish, particularly from Cu, iron, Mn, Ni, and Zn, does not present a threat to human health when consumption is within regulated limits. Furthermore, the study emphasizes the positive impact of soaking treatments, particularly in salt and vinegar solutions, which effectively reduce the HMCs. These treatments help mitigate the potential risks associated with consuming contaminated seafood. This suggests that fish processing techniques, such as soaking, play an essential role in ensuring the safety of marine food products and could be implemented in various settings to enhance public health safety.

#### HQ-HMCs insights

The allowable threshold level of hazard quotient (HQ-HMCs) is one^30^. The HQ-HMCs values for the studied fish species across all consumer groups represented in Table 3. The HQ-Fe values in the fish were varied from 3.80E−02 to 3.80E−01 for sigan; 2.20E−02 to 1.10E−01 for bongos fish; and 1.80E−02 to 1.80E−01 for mallas fish. The HQ-Fe values across all consumer groups are consistently below the permissible limit of 1 and the soaking treatments reduce the Fe levels in the fish, making them safe for consumption across all age groups. The maximum values of HQ-B was recorded for children consumer and the minimum was detected for adults consumer being; 1.50E−01 to 8.20E−01 in sigan fish; 1.70E−01 to 9.60E−01 in bongos fish and 1.60E−01 to 1.10E+00 in mallas fish, all HQ-B values also staying within safe limits except for children who eat mallas fish in control (which exceeds 1, suggesting that the fish in general are mostly safe for consumption following soaking treatments. The maximum values of HQ-Zn was recorded for children consumer and the minimum was detected for adults consumer being; 1.00E−01 to 5.00E−01 in sigan fish; 4.60E−02 to 6.30E−01 in bongos fish and 4.30E−02 to 4.00E−01 in mallas fish, all HQ-Zn values for Zn across the different age groups and species remain within the acceptable range of less than 1, indicating that the levels of Zn in these fish are not a cause for concern for human consumption. The HQ-Mn values in the three fish species, across all age groups, remain well below the permissible limit of 1, suggesting no significant risk from these levels of Mn in the fish. However, it is important to consider the effect of soaking (soak treatment) on the fish, as it could potentially alter these values. The HQ-Mn values ranged from 9.50E−03 to 6.40E−02 in sigan fish, 6.10E−03 to 3.00E−02 in bongos fish, and 6.40E−03 to 3.70E−02 in mallas fish, all significantly below the permissible value, indicating that the levels of Mn are not a concern for human health, even before soaking. The HQ-Ni values were varied from 8.90E−02 to 7.00E−01 in sigan fish, 1.00E−01 to 6.10E−01 in bongos fish, and 9.00E−02 to 6.20E−01 in mallas fish, the HQ-Ni values across all age consumers and species remain within the acceptable range of less than 1, both before and after soaking. This indicates that the levels of Ni in these fish species, even with soaking, do not pose a health risk for human consumption.

**Table 3:** Impact of soaking treatments on hazard quotient (HQ-HMCs) of HMCs in marine fish fillets for children, young, and adults consumers: variation based on different soaking treatments.

	Sigan fish			Bongos fish			Mallas fish		
	Children	Young	Adults	Children	Young	Adults	Children	Young	Adults
HQ-Cu									
Untreated	5.0E−01	1.8E−01	1.0E−01	4.1E−01	1.5E−01	8.5E−02	3.9E−01	1.4E−01	8.1E−02
Salt-soaked	3.1E−01	1.1E−01	6.4E−02	5.1E−01	1.8E−01	1.1E−01	5.2E−01	1.9E−01	1.1E−01
Vinegar soaked	6.1E−01	2.2E−01	1.3E−01	4.4E−01	1.6E−01	9.2E−02	4.9E−01	1.8E−01	1.0E−01
Mixed soaked	4.1E−01	1.5E−01	8.6E−02	3.0E−01	1.1E−01	6.2E−02	3.3E−01	1.2E−01	6.8E−02
HQ-Fe									
Untreated	3.8E−01	1.4E−01	7.9E−02	2.4E−01	8.8E−02	5.0E−02	1.8E−01	6.7E−02	3.9E−02
Salt-soaked	1.4E−01	5.1E−02	2.9E−02	1.4E−01	5.1E−02	2.9E−02	1.3E−01	4.9E−02	2.8E−02
Vinegar soaked	1.8E−01	6.7E−02	3.8E−02	1.6E−01	5.8E−02	3.3E−02	1.3E−01	4.7E−02	2.7E−02
Mixed soaked	1.2E−01	4.5E−02	2.6E−02	1.1E−01	3.9E−02	2.2E−02	8.7E−02	3.2E−02	1.8E−02
HQ-B									
Untreated	7.0E−01	2.6E−01	1.5E−01	8.1E−01	2.9E−01	1.7E−01	1.1E+00	4.1E−01	2.3E−01
Salt-soaked	5.4E−01	2.0E−01	1.1E−01	9.6E−01	3.5E−01	2.0E−01	4.7E−01	1.7E−01	9.8E−02
Vinegar soaked	8.2E−01	3.0E−01	1.7E−01	2.2E−01	8.0E−02	4.6E−02	7.6E−01	2.8E−01	1.6E−01
Mixed soaked	5.5E−01	2.0E−01	1.1E−01	1.5E−01	5.4E−02	3.1E−02	5.1E−01	1.9E−01	1.1E−01
HQ-Zn									
Untreated	9.6E−01	3.5E−01	2.0E−01	6.3E−01	2.3E−01	1.3E−01	4.0E−01	1.5E−01	8.3E−02
Salt-soaked	2.1E−01	7.6E−02	4.4E−02	2.7E−01	9.7E−02	5.5E−02	2.3E−01	8.3E−02	4.8E−02
Vinegar soaked	5.0E−01	1.8E−01	1.0E−01	2.2E−01	8.1E−02	4.6E−02	2.1E−01	7.6E−02	4.3E−02
Mixed soaked	3.4E−01	1.2E−01	7.0E−02	1.5E−01	5.4E−02	3.1E−02	1.4E−01	5.1E−02	2.9E−02
HQ-HMCs-Mn									
Untreated	6.4E−02	2.3E−02	1.3E−02	2.9E−02	1.1E−02	6.1E−03	3.7E−02	1.4E−02	7.7E−03
Salt-soaked	3.0E−02	1.1E−02	6.2E−03	3.6E−02	1.3E−02	7.5E−03	3.0E−02	1.1E−02	6.2E−03
Vinegar soaked	4.6E−02	1.7E−02	9.5E−03	3.0E−02	1.1E−02	6.2E−03	3.1E−02	1.1E−02	6.4E−03
Mixed soaked	3.1E−02	1.1E−02	6.4E−03	2.0E−02	7.3E−03	4.2E−03	2.1E−02	7.5E−03	4.3E−03
HQ-Ni									
Untreated	7.0E−01	2.5E−01	1.5E−01	6.1E−01	2.2E−01	1.3E−01	6.2E−01	2.3E−01	1.3E−01
Salt-soaked	4.2E−01	1.5E−01	8.7E−02	5.1E−01	1.9E−01	1.1E−01	4.7E−01	1.7E−01	9.7E−02
Vinegar soaked	6.4E−01	2.3E−01	1.3E−01	7.4E−01	2.7E−01	1.5E−01	6.5E−01	2.4E−01	1.3E−01
Mixed soaked	4.3E−01	1.6E−01	8.9E−02	4.9E−01	1.8E−01	1.0E−01	4.3E−01	1.6E−01	9.0E−02

#### Overall risk assessment with HI-HMCs values

**Fig. 6:**
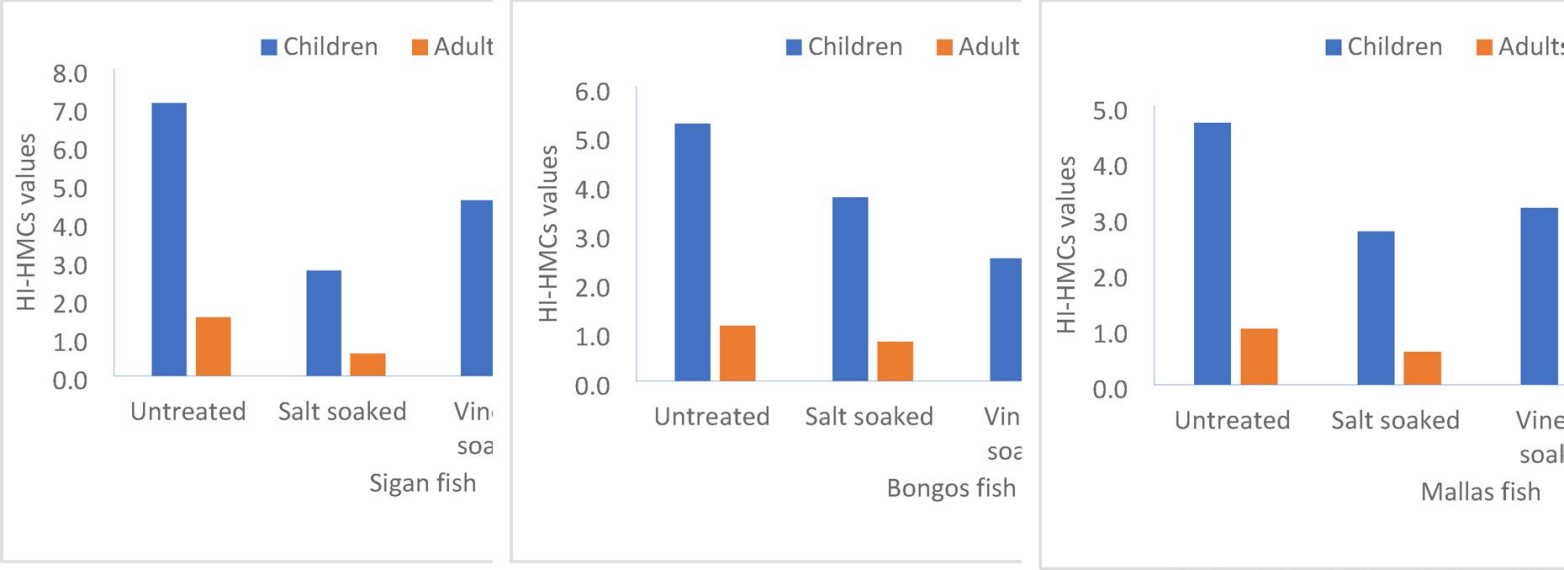
Impact of soaking treatments on hazard index (HI) of HMCs in marine fish fillets from for children, young, and adult consumers: variation based on different soaking treatments.

The Hazard Index (HI) values for HMCs in sigan, bongos, and mallas fish for children, young, and adult consumers after different soaking treatments are presented in Fig. 6. According to the guidelines set by Lei et al., the HI-HMCs values can be interpreted as follows: HI-HMCs ≤ 1: No significant health risks. 1 < HI-HMCs ≤ 10: Potential negative health effects. HI-HMCs > 10.0E+00: Severe or chronic health implications.

For *Sigan fish*, the Hazard Index (HI) values for children indicate potential negative health effects across all treatments, as the values were consistently higher than 1. For instance, the HI-HMCs value in the control sample (C) was 3.30, which exceeds the acceptable threshold for children’s exposure to HMCs, suggesting potential health risks. It is also noted that the HI-HMCs values for children in all soaking treatments (salt-soaked, vinegar soaked, mixed soaked) remained above 1, indicating that HMCs in this species may still pose potential health risks for children. On the other hand, the HI-HMCs values for young and adult individuals were lower than 1 in all treatments, indicating no significant health risks from HMCs exposure in these age groups.

Regarding bongos fish, the HI-HMCs values for children were above 1 in the control sample (C), where the value was 2.74, signifying potential negative health effects. However, after soaking treatments (salt-soaked, vinegar soaked, mixed soaked), the HI-HMCs values decreased but remained above 1 in most cases, suggesting that children are still at risk of potential health effects from the HMCs in this species, despite soaking. For young and adult groups, the HI-HMCs values were below 1 in all treatments, indicating no significant health risks for these age groups. These results emphasize the need for caution when consuming this fish species, particularly for children, while the risk remains minimal for older age groups. For mallas fish, the pattern observed was similar to the other species. The HI-HMCs values for children in the control sample (C) were 2.75, indicating that these values exceed the safe threshold for HMCs exposure and could lead to potential negative health effects. However, there was a noticeable reduction in the HI-HMCs values after soaking treatments (salt-soaked, vinegar soaked, mixed soaked), though the values remained above 1 in most cases. These results suggest that children are still exposed to health risks due to elevated HMCs in this species, even with soaking treatments. For young and adult individuals, the HI-HMCs values were below 1 in all treatments, indicating that there is no significant health risk associated with these age groups’ exposure to HMCs.

The HI-HMCs values indicate that children face potential health risks from HMCs in sigan, bongos, and mallas fish. Although soaking treatments help reduce these risks, the HI-HMCs values remain above 1 in most cases, suggesting the need for caution when children consume these fish. In contrast, young and adult individuals are not exposed to significant health risks, as their HI-HMCs values were below 1 in all cases. Therefore, improving fish processing methods and reducing HMCs, especially for children’s consumption, is crucial.

The observed variation in the HI-HMCs values across the different age groups suggests that children are more vulnerable to the adverse effects of HMCs exposure, particularly from the studied fish. Despite the beneficial effects of soaking treatments, the HI-HMCs values for children often exceeded the safety threshold of 1, indicating that the HMCs in these species may still be a cause for concern. These findings align with the guidelines set by Lei et al., where an HI-HMCs value greater than 1 indicates a potential health risk. In contrast, young and adult individuals were less affected by the HMCs, as their HI-HMCs values remained below 1 across all treatments. This could be attributed to the different metabolic rates and body sizes between children and adults, with children being more susceptible to toxic substances at lower exposure levels. This is a critical finding for public health, especially for populations where children are more likely to consume seafood. Although soaking treatments did reduce the HI-HMCs values for all fish species, it was not sufficient to bring them within the safe limit for children. This suggests that additional measures, such as improved water quality in fish farming, selective harvesting, or the development of more effective soaking or preparation techniques, could be beneficial in further reducing HMCs.

In conclusion, while soaking treatments can help mitigate the risks associated with HMCs exposure, it is imperative to continue exploring safer methods of fish processing, especially for vulnerable populations like children. Further research should also investigate the long-term effects of exposure to HMCs and explore other potential sources of contamination in marine ecosystems.

## Conclusion

This study concludes that the consumption of the three fish species studied is safe in terms of HMCs content, as the EDI-HMCs of Cu, iron, Mn, Ni, and Zn was found to be below the acceptable daily intake (ADI) for each metal. The treatments applied, especially soaking in salt or vinegar solutions, significantly reduced the HMCs in the fish, making them safer for human consumption. However, the soaking treatments can reduce the HI values for HMCs in studied species, but they do not entirely eliminate health risks for children. The HI-HMCs values for children consistently remained above 1 in most cases, suggesting that children may be at potential risk of exposure to harmful HMCs, even after soaking. In contrast, the HI-HMCs values for young and adult individuals remained below 1, indicating no significant health risks. Given that children were found to be the most exposed group, this reinforces the importance of proper treatment of seafood, as they are particularly vulnerable to potential contaminants. Therefore, this study highlights the importance of adopting safe fish-processing methods, including soaking, to mitigate the risks posed by HMCs in seafood and ensures that such fish remain suitable for consumption by all age groups. Further research and policy development are necessary to standardize and promote safe food processing practices to protect public health. Additionally, future studies should investigate the long-term health impacts of HMCs exposure and other potential contamination sources in marine ecosystems.

## Data Availability

The data sets in this study are available from the corresponding author on reasonable request.
